# Preanalytical study concerning the influence of tube type and centrifugation conditions on the concentration of calprotectin in ascites

**DOI:** 10.11613/BM.2026.020703

**Published:** 2026-04-15

**Authors:** Adrijana Dorotić, Marija Siter-Kuprešanin, Helena Čičak, Andrea Saračević, Dora Vuljanić, Rosana Troskot Perić, Igor Alfirević

**Affiliations:** 1 Department of Medical Laboratory Diagnostics, University Hospital “Sveti Duh”, Zagreb, Croatia; 2 Department of Gastroenterology and Hepatology, Referral Center for Functional Gastrointestinal Disorders of the Ministry of Health of Republic Croatia, University Hospital “Sveti Duh”, Zagreb, Croatia; 3 Faculty of Health Studies, University of Rijeka, Rijeka, Croatia; 4 University Department of Surgery, University Hospital “Sveti Duh”, Zagreb, Croatia; 5 Faculty of Dental Medicine and Health, Josip Juraj Strossmayer University of Osijek, Osijek, Croatia; 6 University North, Varaždin, Croatia

**Keywords:** ascites, biochemistry, calprotectin, inflammation, validation/evaluation

## Abstract

**Introduction:**

Concentration of calprotectin in ascitic fluid has been proposed as the possible diagnostic marker for spontaneous bacterial peritonitis. This study aimed to choose the optimal preanalytical conditions for the determination of the concentration of calprotectin in ascitic fluid.

**Materials and methods:**

Study was performed using 20 samples of ascitic fluid of inflammatory etiology. Number of total nucleated (TNC) and polymorphonuclear cells (PMN) were determined on Advia 2120i (Siemens Healthineers). Ascitic samples were collected into three tube types (K2EDTA, tubes with clot activator and Li-heparin tubes) and centrifuged under three different conditions (400xg, 1500xg and 3000xg 15 minutes). Concentration of calprotectin was determined using Bühlmann’s turbidimetric assay and potassium and lactate dehydrogenase using standard laboratory methods on Atellica Solution (Siemens Healthineers). Data was evaluated by Friedman test and Spearman’s correlation coefficient.

**Results:**

The highest concentration of calprotectin was observed in red tubes with clot activator (centrifuged 1500xg 15 minutes; median 0.349 mg/L, IQR 0.094-1.129 mg/L) and the lowest in lavender tubes (centrifuged 3000xg 15 minutes; median 0.109 mg/L, IQR: 0.037-0.850 mg/L). Friedman test showed statistically significant difference between concentration of calprotectin in different preanalytical conditions (P < 0.001). According to the desirable biological variation criteria, differences in calprotectin concentration were clinically significantly different for different preanalytical conditions.

**Conclusions:**

To determine the concentration of calprotectin in ascites, we suggest using a test tube with a red cap and centrifugation conditions of 1500xg 15 minutes which is consistent with the relevant national and international guidelines.

## Graphical abstract

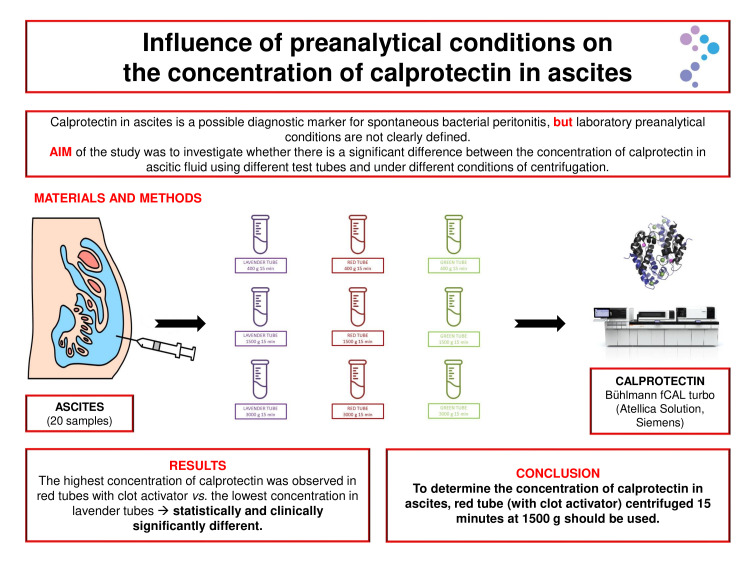

## Highlights

• Calprotectin in ascites is a possible marker of spontaneous bacterial peritonitis

• Preanalytical conditions for calprotectin in ascites are not clearly defined

• For determining calprotectin in ascites, red tube with clot activator (1500xg,15 min) should be used

## Introduction

Peritoneal effusion, widely known as ascites is a pathological accumulation of fluid in the peritoneal cavity due to the increased production or decreased fluid removal. It is a common complication in patients with liver cirrhosis that indicates progression of the disease. In patients with advanced disease stage, ascites accumulation can lead to life-threatening bacterial infection of ascitic fluid, also known as spontaneous bacterial peritonitis (SBP) (*1*-*3*). According to the American Association for the Study of Liver Disease (AASLD) and European Association for the Study of the Liver Disease (EASL) guidelines, the diagnosis of SBP is based on diagnostic paracentesis, the number of polymorphonuclear cells (PMN) in ascites with more than 250x10^6^/L PMN cells and/or a positive finding of ascites culture on exclusively one bacterial strain (*4*, *5*). With the aim to find additional diagnostic marker for SBP, concentration of numerous inflammatory parameters in samples of ascitic fluid, including calprotectin, have been the center of various research studies (*6*-*8*).

Calprotectin is a calcium and zinc-binding protein which is detected almost exclusively in neutrophils. It is secreted or passively released from neutrophils during inflammatory processes and hence it has been investigated as a marker of disease activity in many inflammatory and autoimmune diseases (*9*). Therefore, calprotectin is of particular significance in being accurately reflective of PMN count and their activity in ascitic fluid and has also often been in the center of various research studies as a possible diagnostic and prognostic marker for SBP (*6*-*8*, *10*, *11*).

It has been reported that calprotectin measurement in blood-derived matrices could be affected by preanalytical factors such as sample type, collection and storage conditions (such as temperature), and the presence and type (if any) of used anticoagulants. Some studies have demonstrated higher concentration of calprotectin in serum samples compared to plasma, which makes blood calprotectin concentrations matrix dependent. Although, the dependence of calprotectin concentration on preanalytical factors has been very well investigated in blood-derived matrices and stool samples – commonly used samples in clinical practice, a review of the literature showed that there is no consensus regarding the choice of tube type and centrifugation conditions for determining the concentration of the calprotectin in ascites (*12*, *13*). Considering the dependence of calprotectin on preanalytical conditions in samples derived from blood and stool, our research group hypothesize the same dependence for ascitic fluid samples.

According to the document Laboratory testing of extravascular body fluids: National recommendations on behalf of the Croatian Society of Medical Biochemistry and Laboratory Medicine Part I – Serous fluids, to determine the total and differential cell count in extravascular body fluids, tubes with ethylenediaminetetraacetic acid (EDTA) should be used, while samples of ascites for biochemical analysis should be collected in tubes with heparin as anticoagulant, with the alternative option of using a tube without additives if transport conditions are met (*14*). On the other hand, according to the document of the Clinical and Laboratory Standards Institute (CLSI) C49: A Analysis of Body Fluids in Clinical Chemistry; Approved Guideline, it is recommended that the analysis of body fluids is carried out in the same containers and under the same preanalytical conditions as the comparative blood sample, which is usually tube with red top (*15*).

According to our knowledge, so far, no study has been conducted that examined different preanalytical conditions and their influence on the concentration of calprotectin in ascites. Therefore, the aim of this study was to investigate whether there is a significant difference between the concentration of calprotectin in ascitic fluid using different test tubes and under different conditions of centrifugation. Also, additional aim of the study was to choose the optimal preanalytical conditions for the determination of the concentration of calprotectin in ascitic fluid (using Bühlmann’s turbidimetric assay) for further studies of diagnostic accuracy.

## Materials and methods

### Subjects

The study was performed from June 2023 to February 2024 in the Department of Medical Laboratory Diagnostics, University Hospital Sveti Duh, Zagreb, Croatia. Ascitic fluid samples were collected from patients who were admitted to Emergency Department at University Hospital Sveti Duh, Zagreb, Croatia and Department of Gastroenterology and Hepatology at University Hospital Sveti Duh, Zagreb, Croatia. Inclusion criteria for participation in the study were patients with suspected or advanced liver cirrhosis or malignancy-relates ascites requiring diagnostic paracentesis with abdominal pain, fever, encephalopathy, hypotension, renal failure, acidosis or peripheral leukocytosis. These patients are expected to have a high concentration of leukocytes in ascites, and consequently high number of PMN and therefore elevated concentration of calprotectin. Patients under 18 years of age, as well as patients with traumatic abdominal injuries and a proven intra-abdominal source of infection where not included in the study. The exclusion criteria related to samples included small sample volumes, hemorrhagic, milky appearance or purulent ascites.

Study was approved by the Ethics committee of the University Hospital Sveti Duh. The respondents were familiar with the study procedures and expressed their consent to participate in the study by signing an informed consent form. The study was conducted within the research project „Diagnostic value of calprotectin in early recognition of inflammation“ (KK.01.1.1.04.0055) funded by the European Fund for Regional Development.

### Methods

**Figure 1:**
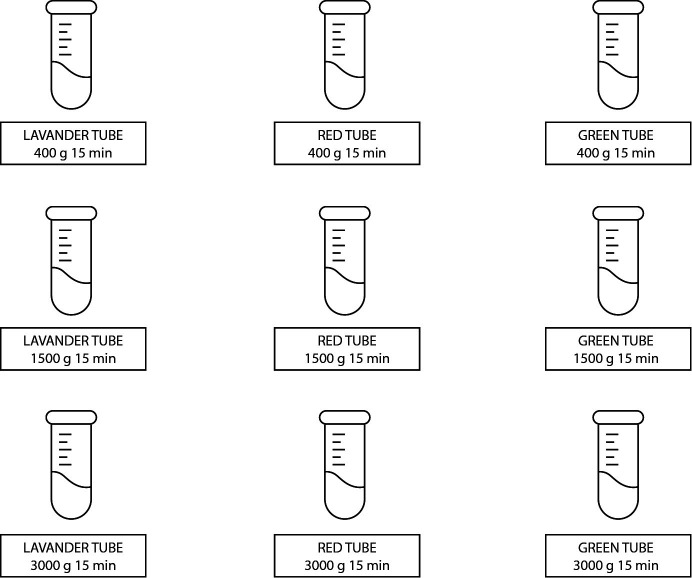
Different preanalytical conditions for testing calprotectin concentration in ascites.

Patients were subjected to diagnostic and therapeutic paracentesis, a simple bedside procedure in which a needle is inserted into the peritoneal cavity and ascitic fluid is being removed. Paracentesis was performed under aseptic conditions from a puncture site in the left or right lower quadrant with the patient in the supine position. All samples were immediately collected at the bedside and processed by laboratory personnel without further delay. Ascitic fluid was immediately transferred into three types of tubes: (*1*) tubes with lavender top that use potassium salt of ethylenediaminetetraacetic acid (K2EDTA) as an anticoagulant which is sprayed onto the interior surface of tube (2 mL, lot 3122664, Becton, Dickinson and Company, Franklin Lakes, USA); (*2*) tubes with red top that use clot activator (4 mL, lot 3086919, Becton, Dickinson and Company, Franklin Lakes, USA) and (*3*) tubes with green top that contain lithium heparin as an anticoagulant (2 mL, lot 3058240, Becton, Dickinson and Company, Franklin Lakes, USA). Each type of tube was filled in triplicate, meaning we had nine tubes in total - three tubes with a lavender top, three tubes with a red top, and three tubes with a green top. All three tube types, in total nine different tubes, containing ascitic fluid were mixed according to the manufacturers’ specifications to ensure thorough mixing of the sample with the additive. Each of the tube type was centrifuged using bench top VWR Mega Star 1.6 centrifuge (VWR International, Radnor, USA), under following different conditions: (*1*) at 400xg for 15 minutes, (*2*) at 1500xg for 15 minutes and (*3*) at 3000xg for 15 minutes. Following the above, nine different preanalytical conditions were taken into the account for each sample, as shown in Figure 1.

Prior to centrifugation, according to the national guidelines for extravascular body fluids, the number of total nucleated cells (TNC) was measured in tubes with K2EDTA anticoagulant using body fluid application on Advia 2120i hematology analyzer (Siemens Healthineers, Erlangen, Germany) (*13*). For the determination of PMN, additionally, cytocentrifuge smear was prepared using cytocentrifuge Rotofix 32A (Hettich Zentrifugen, Tuttlingen, Germany). Cytocentrifugation, a common technique for the capture of cells on microscopic slides, was performed for 8 minutes at 800 rounds *per* minute (RPM), and afterwards, slides were stained in May-Grünwald solution for 3 minutes and Giemsa solution for 12 minutes following the established laboratory protocol. The number of PMN was determined using light microscopy using Olympus BX53 (Olympus, Tokyo Japan), with magnification of 100 times and with immersion oil. To avoid variability in the determination of number of PMN, one experienced specialist in laboratory medicine examined all of the smears and estimated the PMN number. The aforementioned hematology analyzer, Advia 2120i using Body Fluid Application has the ability for the enumeration of TNC count and red blood cells count for pleural, peritoneal, and peritoneal dialysis specimens collected in EDTA tubes. The TNC count is derived from the Basophil/Lobularity channel using BASO reagent containing surfactant and phthalic acid (*16*).

After centrifugation, in all three tube types (nine tubes in total), concentration of calprotectin was determined using automated *in vitro* diagnostic test for the quantitative determination of calprotectin in human stool specimens (Bühlmann fCAL turbo, Bühlmann Laboratories AG, Schönenbuch, Switzerland), with the dilution factor adjusted for ascitic fluid measurements on Atellica Solution automated biochemistry analyzer (Siemens Healthineers, Erlangen, Germany). The aforementioned reagent has been validated and routinely verified by users for the determination of calprotectin concentration in stool samples on Atellica Solution automated biochemistry analyzer. In the manufacturer’s protocol, it is suggested to dilute the stool samples in a ratio of 1:500 with 5 mL of extraction buffer. Subsequently, the raw data from the analyzer is multiplied by a factor of 500 for the mentioned diluted stool extracts in order to obtain the final concentration of calprotectin in stool samples (*17*). Considering that the same dilution was not used in the pre-preparation of ascites samples, and is included in the method application, we applied the division of all results of calprotectin concentration in ascites with a correction factor of 500. A similar protocol was used in the study conducted by Čičak *et al.* to determine the stability of calprotectin concentration with the same reagent on the same analyzer in synovial fluid samples (*18*). Considering the change in sample type, compared to that determined by the manufacturer, and the fact that the protocol has been changed in the context of not diluting samples, the expression of calprotectin concentration has been changed from concentration per sample mass (µg/g) to concentration *per* liter of sample (expressed in SI units as mg/L).

As cell integrity markers, potassium concentration (K) and lactate dehydrogenase (LD) activity were selected, widely known as parameters highly sensitive to cell structural integrity. Under the assumption that during more intense centrifugation force the cellular structures disintegrate and the mentioned markers come out of the cells, by determining their concentration and activity in ascites samples, we wanted to notice subtle differences in K concentrations and LD activity as well as cell disintegration and choose optimal centrifugal force and time. Markers of cell integrity, K concentration and LD activity were determined on the same automatic analyzer. Cells were considered intact at the lowest concentration of K and lowest activity of LD, considering that the mentioned parameters are primarily of intracellular origin, and do not originate from overly aggressive centrifugation of the sample. Concentration of K was measured using potentiometric measurements with integrated indirect multi-sensor technology (IMT) and LD activity was measured using standardized enzymatic method. Due to the structure of K2EDTA anticoagulant, K was not determined in samples collected in K2EDTA tubes. Internal quality control was run daily for all analytes as a part of standard laboratory work. In order to reduce the impact of method imprecision, all biochemistry analytes were measured in duplicate. Also, to reduce the impact of possible instability of the analytes, all preanalytical procedures were made within maximum 10 minutes after sample collection, which is approximate time for transport of the samples to the laboratory.

### Statistical analysis

Statistical analysis was performed using MedCalc statistical software version 12.5.0.0 (MedCalc, Ostend, Belgium). Experiment generated quantitative (numerical) data in paired samples. Since the sample size was relatively small and according to Kolmogorov-Smirnov test for testing the normality of data; they did not follow a normal distribution, a non-parametrical statistical test, Friedman test, was used for testing the difference between the parameters in different preanalytical conditions. For parameters that showed statistically significant difference using Friedman test, *post-hoc* multiple comparison was done. Statistically significant difference was set to 5% and consequently P < 0.05 were considered as statistically significant.

For defining clinically significant difference between concentration of calprotectin in different preanalytical conditions, means of the calculated absolute values of biases were compared with the clinically significant difference criteria. Mentioned criteria was set using biological variation concept. Using the mean value of the biological variability data (*e.g.* within-subject biological variation (CV_I_) and between-subject (CV_G_) biological variation) in females and males for serum calprotectin concentration established by Briers *et al*., and using the formula for calculation the desirable bias (< 0.25 x (CV_i_^2^ + CV_g_^2^)^1/2^) clinically significant difference was set at 14% (*19*). The impact of analytical imprecision was minimized by determining the concentration of all parameters in duplicate and therefore was not considered. The reference point for calculating the bias was the calprotectin concentrations obtained in the red tube centrifuged at 1500xg for 15 minutes because under these conditions the mean calprotectin concentration was highest, and it also corresponds to the conditions under which the accompanying serum sample was centrifuged, as suggested by the CLSI guidelines (*15*).

## Results

Study was performed using 20 samples of ascitic fluid of inflammatory etiology. The results of calprotectin concentrations and other tested biochemistry parameters, as well as cell analysis in different preanalytical conditions are summarized in Table 1. Cell analysis confirmed that some of the samples (N = 4/20) met the diagnostic criteria for SBP (PMN count > 250 x10^6^/L).

**Table 1:** Concentration of calprotectin and other tested parameters in ascites depending on different preanalytical conditions

**Tube type**	**Lavender tube top** **(K2EDTA additive)** **(N = 20)**			**Red tube top** **(clot activator)** **(N = 20)**			**Green tube top** **(lithium heparin additive)** **(N = 20)**		
Centrifugation conditions	400xg 15 min	1500xg 15 min	3000xg 15 min	400xg 15 min	1500xg 15 min	3000xg 15 min	400xg 15 min	1500xg 15 min	3000xg 15 min
Calprotectin (mg/L)	0.121 (0.048- 0.864)	0.162 (0.043- 0.824	0.109 (0.037- 0.850)	0.277 (0.058- 1.076)	0.349 (0.094- 1.129)	0.345 (0.071- 1.014)	0.186 (0.066- 0.900)	0.187 (0.073- 0.715)	0.193 (0.090- 0.802)
Total nucleated cells (x10^6^/L)	162 (69-611)			n.a.	n.a.	n.a.	n.a.	n.a.	n.a.
Polymorphonuclear cells (x10^6^/L)	43 (17-48)			n.a.	n.a.	n.a.	n.a.	n.a.	n.a.
Lactate dehydrogenase (U/L)	68 (58-108)	66 (58-100)	67 (55-109)	68 (60-104)	69 (58-111)	68 (58-115)	70 (59-103)	69 (59-101)	68 (58-115)
Potassium (mmol/L)	n.a.	n.a.	n.a.	3.8 (3.5-4.3)	3.9 (3.4-4.5)	3.9 (3.5-4.5)	3.9 (3.4-4.6)	3.9 (3.4-4.5)	3.9 (3.5-4.5)
Data are expressed as median and interquartile range (IQR). IQR – interquartile range. n.a. – not applicable.									

Results in Table 1 indicate that the highest concentration of calprotectin in ascites is in red tubes that have been centrifuged at 1500xg for 15 minutes (median concentration 0.349 mg/L, IQR: 0.094-1.129 mg/L) and the lowest concentration of calprotectin is obtained in lavender tubes that have been centrifuged at 3000xg for 15 minutes (median concentration 0.109 mg/L IQR: 0.037-0.850 mg/L).

Statistical analysis showed that there is a statistically significant difference between concentration of calprotectin in different preanalytical conditions (P < 0.001) and that there are at least two groups of calprotectin concentrations obtained in different preanalytical conditions statistically significantly different. A more detailed presentation of the preanalytical conditions in which calprotectin concentrations differ significantly is presented in Table 2.

**Table 2:** Testing statistical difference in the concentration of calprotectin in ascites depending on different preanalytical conditions

**Tube number**	**Tube type and centrifugation conditions**	**Statistical difference**	**P-value**
1	Lavender top tube (K2EDTA additive) at 400xg 15 min	(1) *vs*. (5) (6) (9)	P < 0.001
2	Lavender top tube (K2EDTA additive) at 1500xg 15 min	(2) *vs*. (5) (6) (9)	
3	Lavender top tube (K2EDTA additive) at 3000xg 15 min	(3) *vs*. (4) (5) (6) (8) (9)	
4	Red top tube (clot activator) at 400xg 15 min	(4) *vs*. (3)	
5	Red top tube (clot activator) at 1500xg 15 min	(5) *vs*. (1) (2) (3) (7)	
6	Red top tube (clot activator) at 3000xg 15 min	(6) *vs*. (1) (2) (3) (7)	
7	Green top tube (lithium heparin additive) at 400xg 15 min	(7) *vs*. (5) (6)	
8	Green top tube (lithium heparin additive) at 1500xg 15 min	(8) *vs*. (3)	
9	Green top tube (lithium heparin additive) at 3000xg 15 min	(9) *vs*. (1) (2) (3)	
Statistical analysis was performed using Friedman test. P < 0.05 was considered statistically significant.			

For defining clinically significant difference, according to the desirable biological variation criteria, differences in concentration of calprotectin were clinically significantly different for all tested tubes *vs.* red tube centrifuged at 1500xg for 15 minutes. Results of testing clinically significant difference are presented in Table 3.

**Table 3:** Testing clinical difference in the concentration of calprotectin in ascites depending on different preanalytical conditions

**Tube number**	**Tube type and centrifugation conditions**	**Bias (%)**
1	Red top tube (clot activator) at 400xg 15 min	24
2	Red top tube (clot activator) at 1500xg 15 min	0
3	Red top tube (clot activator) at 3000xg 15 min	34
4	Lavender top tube (K2EDTA additive) at 400xg 15 min	45
5	Lavender top tube (K2EDTA additive) at 1500xg 15 min	54
6	Lavender top tube (K2EDTA additive) at 3000xg 15 min	48
7	Green top tube (lithium heparin additive) at 400xg 15 min	30
8	Green top tube (lithium heparin additive) at 1500xg 15 min	26
9	Green top tube (lithium heparin additive) at 3000xg 15 min	35
Absolute mean biases for the calprotectin concentration were calculated for all tube types and centrifugation conditions *vs*. calprotectin concentration in tubes with red top that use clot activator centrifuged at 1500xg for 15 min.		

Statistical analysis indicated that there was no significant difference between the K concentration in the two used tubes types – red top tube *vs*. green top tube regardless of the centrifugation conditions (P = 0.540). On the contrast to the K concentration, there was a statistically significant difference in the LD activity between all tested preanalytical conditions (P = 0.046). Results for statistical analysis of LD activity difference are presented in Table 4.

**Table 4:** Testing statistical difference in the lactate dehydrogenase activity in ascites depending on different preanalytical conditions

**Tube number**	**Tube type and centrifugation conditions**	**Statistical difference**	**P-value**
1	Lavender top tube (K2EDTA additive) at 400xg 15 min		P = 0.046
2	Lavender top tube (K2EDTA additive) at 1500xg 15 min	(2) *vs*. (4) (5) (6)	
3	Lavender top tube (K2EDTA additive) at 3000xg 15 min	(3) *vs*. (4) (5) (6)	
4	Red top tube (clot activator) at 400xg 15 min	(4) *vs*. (2) (3)	
5	Red top tube (clot activator) at 1500xg 15 min	(5) *vs*. (2) (3)	
6	Red top tube (clot activator) at 3000xg 15 min	(6) *vs*. (2) (3)	
7	Green top tube (lithium heparin additive) at 400xg 15 min		
8	Green top tube (lithium heparin additive) at 1500xg 15 min		
9	Green top tube (lithium heparin additive) at 3000xg 15 min		
Statistical analysis was performed using Friedman test. P < 0.05 was considered statistically significant.			

## Discussion

The most significant result of our study is the established statistically and clinically significant difference in calprotectin concentrations in ascites depending on the various test tubes and centrifugation conditions. The highest concentration of calprotectin in ascites was in a red top tubes and the lowest concentration was obtained in a lavender top tube. As expected, the question of further selection of the test tube for determining the concentration of calprotectin in ascites arises.

A review of the literature did not find any studies that attempted to define preanalytical factors for determining calprotectin concentration in ascites. Despite this, various authors try to define diagnostic and prognostic role of calprotectin in ascites for individual clinical entities, very often SBP, without clearly established and referenced preanalytical factors. In study conducted by Fernandes *et al*., ascitic fluid samples were centrifuged during 15 minutes at 3500 RPM, without mentioning the centrifugation force nor collection tube type (*20*). On the other hand, Burri *et al.* centrifugated ascitic fluid samples for 15 minutes at 500xg, also without mentioning the tube type (*21*). Kassem *et al*. in their study stated that ascitic fluid was centrifuged for 15 minutes, but the information about centrifugation force is missing (*22*). Another possibility that has also been observed in the literature is that authors do not mention preanalytical conditions at all, as in the study of the Egyptian authors Abdel Rahman *et al*. (*8*). Given the fact that our study showed a clear influence of the mentioned preanalytical factors on the concentration of calprotectin in ascites, the need to standardize and to understand these conditions for further studies of diagnostic accuracy is clearly justified.

The observed differences in calprotectin concentrations in different tube types can be explained by differences in the molecular structures of the additives in individual tubes. EDTA anticoagulant has the ability to chelate divalent cations, and therefore also chelate calcium and zinc ions, which are crucial for the optimal structure and consequently also for the function of the calprotectin molecule. Studies of structural analysis, performed by Imani *et al.,* of calprotectin molecule with fluorescence spectroscopy which included alteration in concentration of calcium, zinc and manganese, showed a remarkable change in secondary and tertiary structure of tested protein depending on the metal ions (*23*). Therefore, a result showing a significantly statistically and clinically lower concentration of calprotectin in a tube which have the ability to chelate crucial metal ions, is not surprising. Considering presented results; authors strongly discourage the use of lavender top tubes in determining the concentration of calprotectin in ascitic fluid samples. Similar to our results, but analyzing a different sample type, in their preanalytical study, Pedersen *et al.* found significantly higher calprotectin concentrations in serum and Li-heparin plasma samples *vs.* EDTA samples (*24*). The explanation for the differences in calprotectin concentrations obtained in red and green tubes may be found in the sensitivity of neutrophils to the blood clotting process. Calprotectin is detected almost exclusively in neutrophils that are *in vitro* highly dependent on platelet activation and coagulation process which may induce release of intracellular calprotectin into the extracellular medium and lead to higher concentrations of calprotectin in serum, as proposed by study conducted by Honap *et al.* (*25*).

According to our results, there is no statistically significant difference between the K concentration, in the different preanalytical conditions. Lactate dehydrogenase activity has been the lowest in the lavender tube top centrifuged for 15 minutes at 1500xg; indicating that these centrifugation conditions affect cell structure to the smallest extent. In their short communication, Veena *et. al* showed that K and LD levels in serum were found to be raised by increasing the centrifugal force up to 3000xg (*26*).

Considering the highest calprotectin concentration in ascites was observed in red tube and the lowest LD activity with the centrifugation conditions of 15 minutes at 1500xg, authors suggest using red tube centrifuged for 15 minutes at 1500xg for determining the concentration of calprotectin in ascites.

A possible limitation of our study could be a relatively small number of samples included in the study. The authors believe that the nature of the samples used justifies their small number. A more significant possible limitation of the study concerns the fact that most of the samples included in the study did not meet the criteria for diagnosing SBP and the obtained calprotectin concentrations are relatively small (smaller than in similar studies). Question arises whether the same results would be extrapolated to the higher calprotectin concentrations.

On the other hand, there are numerous novelties of our study. Presented study was performed on an automated biochemical analyzer using an automated method that significantly speeds up the time required for making laboratory test results unlike most literature examples that report the use of ELISA or point of care tests for determining the calprotectin concentration in ascites. Also, automated method is not subject to variability related to laboratory personnel, as is the case with smear examination, which is still the suggested diagnostic method in case of SBP assessment. According to our knowledge, this kind of preanalytical study in ascitic fluid samples has not been conducted yet. The proven influence of different preanalytical conditions on the concentration of calprotectin in ascitic fluid samples once again irrefutably confirms the importance of preanalytical processes in laboratory medicine. This study provides comprehensive analysis of different preanalytical conditions and their influence on the concentration of calprotectin in ascites for future studies.

Extravascular body fluids are a relatively unexplored area in the laboratory medicine and therefore provide wide possibilities in the diagnostic field in the future. However, before being introduced into the routine clinical practice, these new sample types require the satisfaction of clearly set quality criteria in the area of method validation. Clearly defining the basic preanalytical conditions, such as the type of test tubes and centrifugation conditions, are one of them. We would like to emphasize that there is a significant difference between the concentration of calprotectin in ascitic fluid using different test tubes and under different conditions of centrifugation. In conclusion, considering the observed difference between the concentration of calprotectin in various preanalytical conditions, to determine the concentration of calprotectin in ascites, we suggest sampling in a test tube with a red cap and centrifugation conditions of 15 minutes and 1500xg which is in accordance with relevant national and international guidelines (*14*, *15*).

## Data Availability

The data generated and analyzed in the presented study are available from the corresponding author on request.
